# Usefulness of a New Large Set of High Throughput EST-SNP Markers as a Tool for Olive Germplasm Collection Management

**DOI:** 10.3389/fpls.2018.01320

**Published:** 2018-09-21

**Authors:** Angjelina Belaj, Raul de la Rosa, Ignacio J. Lorite, Roberto Mariotti, Nicolò G. M. Cultrera, Carmen R. Beuzón, J. J. González-Plaza, A. Muñoz-Mérida, O. Trelles, Luciana Baldoni

**Affiliations:** ^1^IFAPA Centro Alameda del Obispo, Córdoba, Spain; ^2^CNR – Institute of Biosciences and Bioresources, Perugia, Italy; ^3^Instituto de Hortofruticultura Subtropical y Mediterranea, Universidad de Málaga-Consejo Superior de Investigaciones Científicas, Málaga, Spain; ^4^CIBIO, InBIO – Research Network in Biodiversity and Evolutionary Biology, University of Porto, Porto, Portugal; ^5^Department of Integrated Bioinformatics, National Institute for Bioinformatics, Universidad de Málaga, Málaga, Spain

**Keywords:** Olea europaea, germplasm collection, identification, oleasters, EST-SNPs

## Abstract

Germplasm collections are basic tools for conservation, characterization, and efficient use of olive genetic resources. The identification of the olive cultivars maintained in the collections is an important ongoing task which has been performed by both, morphological and molecular markers. In the present study, based on the sequencing results of previous genomic projects, a new set of 1,043 EST-SNP markers has been identified. In order to evaluate its discrimination capacity and utility in diversity studies, this set of markers was used in a representative number of accessions from 20 different olive growing countries and maintained at the World Olive Germplasm Collection of IFAPA Centre ‘Alameda del Obispo’ (Córdoba, Spain), one of the world’s largest olive germplasm bank. Thus, the cultivated material included: cultivars belonging to previously defined core collections by means of SSR markers and agronomical traits, well known homonymy cases, possible redundancies previously identified in the collection, and recently introduced accessions. Marker stability was tested in repeated analyses of a selected number of accessions, as well as in different trees and accessions belonging to the same cultivar. In addition, 15 genotypes from a cross ‘Picual’ × ‘Arbequina’ cultivars from the IFAPA olive breeding program and a set of 89 wild genotypes were also included in the study. Our results indicate that, despite their relatively wide variability, the new set of EST-SNPs displayed lower levels of genetic diversity than SSRs in the set of olive core collections tested. However, the EST-SNP markers displayed consistent and reliable results from different plant material sources and plant propagation events. The EST-SNPs revealed a clear cut off between inter- and intra-cultivar variation in olive. Besides, they were able to reliably discriminate among different accessions, to detect possible homonymy cases as well as efficiently ascertain the presence of redundant germplasm in the collection. Additionally, these markers were highly transferable to the wild genotypes. These results, together with the low genotyping error rates and the easy and fully automated procedure used to get the genotyping data, validate the new set of EST-SNPs as possible markers of choice for olive cultivar identification.

## Introduction

Olive, the iconic Mediterranean fruit tree species, owns a very rich genetic patrimony, represented by a very high number of cultivars, wild trees and related subspecies. The reasons leading to such a high genetic variability are mainly related to its allogamous nature, a lack of turnover with modern cultivars, a remarkable tree longevity and a good capacity to survive without cultivation (Baldoni and Belaj, 2009; Beghè et al., 2011; Belaj et al., 2016). Each country or region of the Mediterranean Basin and beyond, characterized by a wide range of environments and growing systems, has maintained its own local and traditional cultivars (Barranco and Rallo, 2000; Marra et al., 2013). Nowadays, the cultivated germplasm is estimated to include more than 1,200 clonally propagated cultivars, that are still under cultivation or have been collected, propagated and conserved in over 100 *ex situ* field collections (Bartolini, 2008), with the aim to protect the olive genetic patrimony of all olive growing countries and to ensure conservation against potential risks of extinction. National and international collections represent essential tools to avoid or minimize the loss of traditional local cultivars and to use for any future breeding program (Caballero et al., 2006; Belaj et al., 2016). In this sense, three World Olive Germplasm Banks (WOGB) were established in Córdoba (Spain), Marrakech (Morocco), and Izmir (Turkey), aimed at acquiring, maintaining, evaluating, and making accessible as much genetic diversity of the species as possible.

The management of germplasm collections represents a complex task, due either to the above mentioned high variability of the olive germplasm, as well as to the scattered and partial identification and characterisation of olive cultivars and to the confusion on their denominations (Belaj et al., 2004b, 2016; Baldoni et al., 2009; Haouane et al., 2011; Mousavi et al., 2017b). A functional olive germplasm collection requires an efficient strategy to incorporate new accessions, while ensuring homogeneity and authenticity of cultivars, discarding, or reducing to minimum redundant genotypes (Belaj et al., 2003a; Atienza et al., 2013; Trujillo et al., 2014). Thus, the identification of plant material is crucial and represents the first step toward a correct germplasm management.

Most of the current identification efforts in plant germplasm collections are based on DNA markers. In this sense, an adequate molecular marker system for olive identification should fulfill the following desirable properties: availability of many polymorphisms, codominant inheritance, high frequent occurrence, easy accessibility, low cost, quick and high throughput, high reproducibility and transferability among different laboratories and detection platforms (Bracci et al., 2011; De Lorenzis et al., 2015; Mason et al., 2015; Marrano et al., 2017).

Olive genotyping is currently based on microsatellite markers (SSR), that have shown high efficiency for the identification of olive cultivars coming from olive groves (Beghè et al., 2011; Rehman et al., 2012; Fernández et al., 2015), or from regional (Marra et al., 2013; Mousavi et al., 2017b), national (Fendri et al., 2010; Chalak et al., 2014; Muzzalupo et al., 2014) and international collections (Sarri et al., 2006; Haouane et al., 2011; Trujillo et al., 2014). These markers have largely superseded the traditional morphological description (Barranco and Rallo, 2000; Caruso et al., 2014; Messora et al., 2017) and other major molecular techniques (RFLP, RAPD and AFLP) used in olive (Belaj et al., 2003b, 2016; Bracci et al., 2011). Up to date, SSRs have been the markers of choice in olive due to their high level of polymorphism, multi allelic and codominant nature and the high reproducibility within laboratories. However, microsatellite markers present some main drawbacks related to the wide use of dinucleotide loci, that give rise to very close in size neighboring alleles, making thus difficult their distinction and separation during allele binning (Baldoni et al., 2009; Trujillo et al., 2014). Thus, comparison and convergence among different laboratories and within large SSR datasets is not easy, and size discrepancies for the same allele may cause miscalling and generate confusions (Baldoni et al., 2009; Trujillo et al., 2014; Beghè et al., 2015). The recent development of long core repeat SSRs (de la Rosa et al., 2013; Mariotti et al., 2016; Arbeiter et al., 2017), based on EST sequences of transcriptome collections (Alagna et al., 2009, 2016; Muñoz-Mérida et al., 2013; Shah et al., 2017), displaying higher allelic separation and more precise allele calling, could help to solve some of the above-mentioned problems. However, their low polymorphism would probably increase the total number of markers required for genotype characterisation and identification.

In recent years, special attention is being paid to the development and use of SNP (Single Nucleotide Polymorphism) markers in olive (Reale et al., 2006; Consolandi et al., 2007; Muleo et al., 2009; Hakim et al., 2010; Belaj et al., 2012; Dominguez-Garcia et al., 2012; Kaya et al., 2013; Biton et al., 2015; Ipek et al., 2016). SNP markers show several advantages over SSRs, because they are very abundant along the genome, display a low mutation rate, may be applied at high-throughput scale (1000s of markers from each run) and may be screened at a single centralized platform. Besides, SNPs are sequence-based and distinguished according to the nucleotide present at each given position, which confers them high reproducibility among laboratories and detection techniques (Bracci et al., 2011; Bevan et al., 2017). For these reasons, they could be used to accurately genotype large sets of genotype accessions, allowing for a reliable interchange and comparison of independent datasets among collections. Furthermore, SNPs derived from EST sequences may play a functional role, to uncover the genetic bases of their agronomical performance (Kaya et al., 2013; Biton et al., 2015; Ipek et al., 2016). However, despite the above-mentioned advantages, until recently the number of SNP markers available for olive was very limited and only a few recent studies have reported large-scale SNP subsets to use for genetic mapping (Ipek et al., 2016), as well as for genetic diversity and relationship studies (Kaya et al., 2013; Biton et al., 2015).

Based on olive transcriptomic data derived from OLEA and OLEAGEN genomic projects (Muñoz-Mérida et al., 2013; Muleo et al., 2016), we intended to create a large set of SNP markers, representing a new tool for olive cultivar precise identification and, as a consequence, to improve olive collections management. The new set of EST-SNPs was used to genotype the olive accessions from the World Olive Germplasm Collection of IFAPA (Córdoba, Spain), which is currently one of the largest olive germplasm banks (Belaj et al., 2016) and counts on a long experience on olive cultivar characterisation by means of morphological (Barranco et al., 2005; Trujillo et al., 2014) and molecular descriptors (Belaj et al., 2001, 2004b,c; Atienza et al., 2013; Trujillo et al., 2014). We intended to cover, for the first time by means of EST-SNPs, the most important aspects of the management of the olive germplasm collections. For that purpose, particular attention has been paid to the evaluation of: (1) the reproducibility of the of the new EST-SNP markers, (2) the ability of these markers to recognize redundant genotypes, identity errors, possible intracultivar variation and identification of different accessions, (3) testing the transferability of this marker set into the wild germplasm and (4) establishing the level and distribution of EST-SNP diversity in a representative sample of olive cultivars and wild trees.

## Materials and Methods

### SNP Mining, Selection and Genotyping

In the framework of OLEAGEN^1^ and OLEA^2^ projects, data from transcript libraries derived from fruits, at different stages of development, from high- and low-phenolics varieties (Alagna et al., 2009), and from leaves, roots and buds of other varieties (Muñoz-Mérida et al., 2013), were obtained by pyrosequencing technology. Before SNP search, a cleaning step was performed using the open-source tool Seqclean^3^, to discard contaminant sequences as vectors and primers used in generating the cDNA libraries.

Identification of SNPs in the libraries was carried out using the sequence search and alignment software ssahaSNP (Ning et al., 2005). The output file containing the SNPs was parsed and information saved as tab-separated values (TSV format).

An additional search was carried out using QualitySNP software (Tang et al., 2006). In brief, the original cleaned reads were used as a query and QualitySNP pipeline carried out an assembly through CAP3 module and a posterior search for SNPs. One of the output files contained the most probable SNPs, the reads in which they are located and the location info for each polymorphism, including nucleotide changes. The criteria applied for SNP discovery were: designability score higher than 0.6, minimum distance of 50 bp to other SNPs in the same contig, at least eight reads in the contig with a minimum of four ESTs, and two reads of the least frequent allele. The identification of these SNPs made possible the selection of a large subset of markers for olive genotyping by using an Illumina Golden Gate platform at the Human Genotyping Unit-CEGEN of the Spanish National Cancer Research Centre (CNIO, Madrid, Spain). The selection of the subset SNP markers was based on their amplification success in a representative sample of 36 cultivars, as well as applying the following criteria: GenTrain score higher than 0.5, call frequency higher than 0.9, minor allele frequency higher than 0.06, heterozigosity excess lower than 0.8 and call rate higher than 0.8. These criteria were applied using the software Genome Studio 2008.1 (Illumina, San Diego, CA, United States). In addition, SNP markers with more than 22 missing data were excluded from further analysis.

A total of 5,154 SNPs was identified from 2,733 contigs. From this set of SNPs, 3,000 were randomly chosen for amplification in a representative sample of 36 olive cultivars, being 2,399 those with a consistent amplification. Among them, only a set of 1,043 loci fulfilled all the above-mentioned selection criteria and were used for further genotyping studies (**Supplementary Table 1** and PRJEB27972). The olive accessions were SNP genotyped by means of the Golden Gate assay performed on the Illumina’s Bead Array TM at the Human Genotyping Unit-CEGEN of the Spanish National Cancer Research Centre (CNIO, Madrid, Spain). All the results referred in this study are based on the analysis of the final set of 1,043 markers.

### Plant Material

To evaluate the suitability of the new EST-SNP markers for cultivar identification, diversity studies, and their transferability into the wild germplasm, several cultivated and wild genotypes were included in the study, as well as some seedlings from an inter-varietal cross progeny (**Table 1** and **Supplementary Tables 2**, **3**). Total DNA was extracted from fresh leaves of each sample following the protocol described by de la Rosa et al. (2002).

**Table 1:** Number of samples analyzed for cultivars, wild plants and genotypes from breeding progenies.

Type of material	Number of samples	Range of allelic differentiation
(A) Cultivated material^∗^	325	
Duplicated samples	60	0
Different trees/accession	33	0
Different accessions sharing the same name and identity^∗∗^	26	0
Previously identified redundancies	75	0–10
New and not previously identified accessions^∗∗∗^	69	
Different cultivars	156	200–680
Homonymy groups	16	200–680
(B) Genotypes from a cross ‘Picual’ x ‘Arbequina’ cultivars	15	200–680
(C) Wild genotypes	89	200–690


*^∗^Some of the samples are shared in different groups. ^∗∗^Accessions introduced at different moments in the collection but sharing the same names and previously identified as belonging to the same cultivar. ^∗∗∗^Within the new introduced accessions, different cultivars with expected allelic ranges of differentiation (200–680) were found, as well as redundancies without any EST-SNP differentiation.*

The cultivated plant material comes from the World Olive Germplasm Collection located at IFAPA, Centre Alameda del Obispo (Córdoba, Spain), established more than 45 years ago. This collection represents the first international attempt of conservation and management of the olive germplasm and was supported by the International Olive Council (IOC) through a FAO-INIA project (Caballero et al., 2006). At present, coordinated by a network of institutions of Andalusia (Southern Spain), providing new resources and facilities, the collection accounts about 900 accessions from 26 countries (Belaj et al., 2016), more than half being already characterized and identified by means of molecular markers and/or morphological descriptors (Belaj et al., 2004b, 2012; Atienza et al., 2013; Trujillo et al., 2014). All olive accessions are grown in the field with two or three replicates (trees). Each cultivated accession is provided by a unique and never reused collection register number.

In order to validate the utility of the new EST SNP markers for the unambiguous identification of olive germplasm collections and trying to clarify the most important issues for their effective management, a total of 325 cultivated samples were selected for the EST-SNP analysis. The cultivated plant material comprise olive accessions from 20 different olive growing countries (**Supplementary Table 2**) and it included: (a) 60 technical duplicates from a total of 28 accessions, resulted from at least two independent DNA extractions of the same tree; (b) 15 accessions which were represented by more than one tree (33 samples); (c) 26 accessions introduced at different moments in the collection, but sharing the same names and found, in a later stage, to belong to the same cultivars by means of different molecular markers (Atienza et al., 2013; Trujillo et al., 2014) d) other redundancies (75 accessions) displaying the same genotype to other cultivars in the collection, and which were previously identified by means of morphological descriptors, RAPD, DArT and SSR markers (Belaj et al., 2004b; Barranco et al., 2005; Atienza et al., 2013; Trujillo et al., 2014) (e) 16 well known homonymy cases, i.e., the same names used for different cultivars; as well as (f) 69 new and not previously identified accessions which were planted recently (2012) at the WOGB IFAPA field collection. Introduced to the collection within the frame of RESGEN projects, the new accessions came from different regional Italian donor collections. Besides, (g) a representative sample of the entire cultivated olive diversity, corresponding to the cultivars included in different core collections sets of WOGB IFAPA (Belaj et al., 2012) was included in the study (**Supplementary Table 2**). Each core set consisted in increasing numbers of cultivars: 18, 27, 36, 45, or 68, respectively representing from 5 to 19% of the whole collection). The EST-SNP genotyping of cultivated material also included (h) other economically important cultivars coming from all over the Mediterranean basin.

In addition to the cultivated material, a set of 89 wild olive genotypes (*Olea europaea* subsp. *europaea* var *sylvestris*), from the *ex situ* Wild Olive Repository of IFAPA, Córdoba (Belaj et al., 2011; de la Rosa et al., 2014), were also included in the study (**Supplementary Table 3**). Most of these wild genotypes (73) came from prospecting surveys in oleaster forests of different Spanish areas (Balearic Islands, Andalusia and Extremadura regions). Besides, 16 genotypes which belong to *O. europaea* subsp. *guanchica*, originally from Canary Islands, were also included in the present study. Finally, 15 genotypes from a cross ‘Picual’ x ‘Arbequina’ cultivars from the IFAPA olive breeding program (León et al., 2007) were also genotyped by the EST-SNP markers.

### Data Analysis

Pairwise multilocus matching was applied within the entire cultivated set of samples, in order to measure the distance between each pair by using the GenAlex 6.5 software (Peakall and Smouse, 2012). Key genetic parameters were separately calculated on non-redundant cultivated and wild genotypes. The following parameters were analyzed: Average Number of Alleles (N_avg_), Number of Effective Alleles (N_e_), Minor Allele Frequency (MAF), Shannon’s Information Index (I), Observed (H_o_) and Expected Heterozygosity (H_e_), and Fixation Index (F). Cervus v.3.0.7 software (Marshall et al., 1998; Kalinowski et al., 2007) was used to calculate the Polymorphic Information Contents (PIC) and the Estimated Frequency of Null Alleles (F_Null_) for each EST-SNP locus.

The diversity values found for the set of EST-SNP markers in the core collections were compared with those obtained by the 32 SSRs used for their development (Belaj et al., 2012). Pairwise genetic distances as defined by Peakall and Smouse (2012) were computed using the Distance procedure implemented in GenAlEx 6.5 to assess the relationships among cultivated varieties, as well as their relationships with the wild germplasm. The genetic distance matrix, constructed by GenAlEx, was subjected to the analysis of molecular variance (AMOVA) approach (Excoffier et al., 1992) using the same program. Pairwise comparisons between wild olive populations and local cultivars examined with AMOVA resulted in values of *ϕ_st_* that were equivalent to the proportion of the total variance that is partitioned between two populations/groups.

The evolutionary history inferred by the Neighbor-Joining method using the MEGA7 software (Kumar et al., 2016), was performed both on the total cultivated samples including replicates and on the unique cultivated genotypes, wild types and crossbred progenies. The percentage of replicate trees in which the associated taxa clustered together in the bootstrap test (500 replicates) are shown next to the branches. The round dendrogram with unique genotypes, wild types and crossbred progenies was visualized by using Figtree software^4^.

## Results

### Evaluation of EST-SNPs Utility for a Reliable and Effective Management of an Olive Germplasm Collection

**FIGURE 1:**
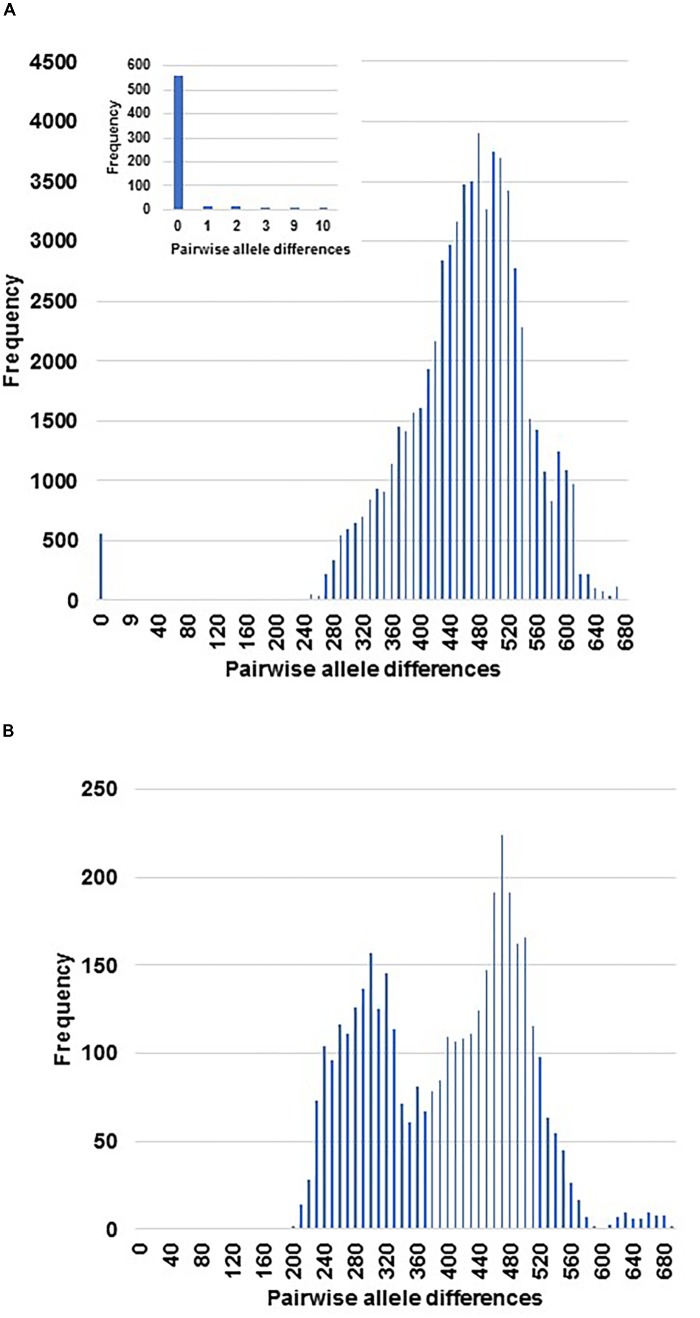
Representation of the genetic distances among the entire cultivated sample set and the genotypes from the cross ‘Picual’ x ‘Arbequina’ cultivars **(A)** and the wild genotypes **(B)**. The distances were measured in number of different alleles for the pairwise comparisons among the set of samples under study with 1043 EST-SNPs. The small window is a zoom for the smallest distance zone among cultivated samples.

The 1,043 EST-SNP markers used to analyze the set of 325 cultivated samples under study, gave rise to 52,650 pairwise comparisons. The histogram constructed on distances proportional to the number of different alleles for all comparisons, showed two parts which were clearly separated by a large gap between them (**Figure 1A**). Thus, the left side of the histogram included a small part of the pairwise comparisons (605 pairs, representing around 1.2% of the total) displaying the lowest allelic differentiation (from 0 to 10 different alleles), i.e., only up to 0.005% of the total number of the EST-SNP alleles. While the pairwise comparisons displaying high allelic differences (from 200 to more than 680 alleles) for each pair of accessions, were included at the right side of the histogram. A high percentage (92%) of the pairwise comparisons included at the left side of the histogram were characterized by lack of allelic differentiation between them (**Figure 1A**). This is the case for all duplicated samples (60 from 28 different accessions), resulting from independent DNA extractions from the same tree (**Table 1** and **Supplementary Table 2**).

The EST-SNP genotyping did not reveal any allelic differences between different trees belonging to the same accessions (33). Similarly, no allelic differentiation was found between the accessions (26) distinguished by different register numbers, because of their introduction at different moments in the collection, but sharing the same names and same molecular profiles, according to previous identification studies (**Table 1** and **Supplementary Table 2**). Overall, independent DNA extractions from the same tree as well as from different trees and accessions of the same cultivar fully matched at all genotyped SNP loci, confirming thus the reliability and the stability of this new set of markers for olive cultivar genotyping.

All the redundant genotypes, either the ones described previously in the collection as well as the new redundancies identified in the present study, were also found at the left side of the histogram (**Figure 1A** and **Supplementary Table 4**). Thus, the pairwise comparison of all accessions made possible the detection of 59 redundancy groups with two or more cultivars/accessions, encompassing a total of 164 cultivated samples with identical EST-SNP profiles (**Supplementary Table 4** and **Supplementary Figure 1**). Among them, the largest group of redundancies was that of the main Italian cultivar ‘Frantoio,’ including 7 identical accessions (‘Augellina,’ ‘Corsicana da Olio,’ ‘Larcianese,’ ‘Oblonga’ ‘Frantoio A. Corsini,’ ‘Kokerrmadh Berati,’ and ‘Aggezi Shami-1’). Whereas ‘Maelia,’ and ‘Razzola’ displayed very little allelic differentiation to ‘Frantoio’ (1 and 2 alleles, respectively) and between them (3 alleles).

The same EST-SNP profiles were obtained in all cases of previously described synonymies (i.e., different naming for the same cultivar). For instance, no EST-SNP’s differentiation was found within a group of cultivars from neighboring countries such as ‘Alameño de Marchena’ and ‘Cañivano Blanco’ from Spain, ‘Picholine Marocaine,’ ‘Haouzia’ and ‘Menara’ from Morocco, as well as the Algerian cultivar ‘Sigoise.’ The same was found for the redundancy group formed by the Syrian ‘Abou Choki-1126,’ the Cypriot ‘Athalassa’ and the Lebanese ‘Beladi’ cultivars (**Supplementary Table 4** and **Supplementary Figure 1**). Identical profiles were also observed among the recently introduced and not previously identified accessions, sharing 38 of them the same SNP genotype with at least another accession. New cases of identity among cultivars were highlighted, such as the group of accessions ‘Confetto,’ ‘Manna,’ ‘Nera di Gonnos,’ ‘Sivigliana da Mensa,’ ‘Maiorca’ and ‘Nocellara Messinese,’ as well as the pair of accessions ‘Rossellino’ and ‘Rosino.’ In addition, some of the newly introduced accessions shared the same genotype with other cultivars from different countries, previously introduced in the collection (**Supplementary Table 4** and **Supplementary Figure 1**). This is the case of the pairs of accessions ‘Bianchera’ – ‘Istarska Bjelica’ (Italy – Croatia, respectively), ‘Grossa di Spagna’ – ‘Gaydorelia’ (Italy – Greece), as well as the group formed by the cvs. ‘Bella di Spagna’ and ‘Santa Caterina’ with ‘Gordal Sevillana’ (Italy – Spain).

In additional, identical EST-SNP profiles were also found for synonymy cases which had been described as potential somatic mutations cases in previous identification studies (**Supplementary Table 4**). And that included either probable mutation cases without effect on the phenotype traits (as in the case of cv ‘Frantoio’ and ‘Frantoio A. Corsini’), as well as those with modifications in the leaf morphology (‘Picual de Hoja Oscura’ and ‘Picual de Hoja Clara’ versus the cultivar ‘Picual’), fruits and even agronomic traits (‘Zarza’ and ‘Lechín de Sevilla’). While very slight allelic differentiations were displayed in the cases of the pairs ‘Picudo’ - ‘Picudo de Fruto Rojo’ and ‘Dulzal’ – ‘Manzanilla de Sevilla,’ with 9 and 10 different alleles out of 2,086 (0.0044–0.0049%), respectively.

The EST-SNP markers under study did not display any allelic differentiation among the set of morphologically similar cultivars ‘Meloncillo’ – ‘Ojua’ – ‘Mollar de Cieza’ – ‘Verdalon,’ as well as the pair of cultivars ‘Negrillo de Estepa’ – ‘Alameño de Cabra,’ maybe because of possible prospecting redundancies. In line with this, the same EST-SNP genotypes were found for cultivars collected in the same or very close geographic areas, such as the pairs of cultivars ‘Escarabajuelo de Ubeda’ – ‘Jabaluna’ and ‘Nevadillo Blanco de Lucena’ – ‘Tempranillo de Lucena,’ from Spain. Similarly, the recently introduced accessions ‘Palma’ and ‘Filare’ shared the same EST-SNP genotype with two well-known cultivars from the same or nearby geographic area (‘Bosana’ and ‘Moraiolo,’ respectively).

This set of EST-SNP markers confirmed other cases of previously identified redundancies due to possible errors in different stages of plant material conservation and management. This was the case of trees of the cultivars ‘Tanche,’ ‘Hamed,’ ‘Adkam,’ which grouped together with ‘Itrana,’ ‘Manzanilla de Sevilla’ and ‘Shami’ groups, respectively. Possible management errors may also occur during the introduction of new germplasm into the collection. Thus, the finding of the same EST-SNP profile for the pair of the recently introduced accessions ‘Ravece’ and ‘Rotondella’ may be due to propagation and/or planting errors, as well as possible donor collection’s mistakes (**Supplementary Tables 2**, **4**).

Overall, the use of the set of 1,043 EST-SNP markers for genotyping of the samples under study made possible the identification of 156 non-redundant cultivated genotypes which showed a high allelic differentiation between them. As expected, all previously well-known homonymy cases (the same generic name for different cultivars) such as ‘Abbadi,’ ‘Manzanilla,’ ‘Toffahi,’ ‘Carrasqueño’ (**Supplementary Table 5**) were included among the non-redundant genotypes. In addition, similar allelic differentiation with the non-redundant cultivated genotypes were also found for the 15 genotypes from the cross ‘Picual’ x ‘Arbequina’ (**Figure 1A**) and the wild genotypes (**Figure 1B**). These results may indicate a seedling origin of the non-redundant accessions of our collection.

### Genetic Diversity and Relationships Within and Among Cultivated and Wild Genotypes

All new EST-SNP markers resulted polymorphic in the non-redundant cultivars identified in the present study. The mean number of Ne per locus was 1.53 (**Supplementary Table 6**). In general, a relatively wide variability was found for the genetic parameters determined in this group of cultivars. Thus, minor allele frequency (MAF) values ranged from 0.02 to 0.50 in non-redundant cultivars, with an average of 0.24 (data not shown). A total of 41.6% of the EST-SNP marker set displayed MAF values between 0.30 and 0.50. Ho values ranged from 0.03 to 0.994, averaging 0.37, while He ranged from 0.04 to 0.50, with mean values of 0.32. Shanon diversity index (I) values ranged from 0.09 to 0.69, being the mean value of 0.49. PIC values ranged from 0.04 to 0.37 with mean values of 0.26; only 48 EST-SNPs showed PIC values below 0.1, while 75% showed values higher than 0.2. The mean average fixation index (F) was -0.123, ranging from -0.987 to 0.487. Most of the EST-SNPs did not diverge or showed not significant deviation from Hardy–Weinberg equilibrium.

Both previously reported SSR and the EST-SNP markers analyzed here, when compared in different core cultivar sets, were informative (**Table 2**). However, as expected, higher diversity values were found for the SSR markers. The average EST-SNP effective number of alleles did not vary at all core levels (1.54–1.55), whereas higher and relatively wider ranges were found for SSRs (3.96 and 4.36 for cores 18 and 36, respectively). Similarly, the mean H_o_ and H_e_ based on SSR markers displayed higher values than those calculated for EST-SNP markers. The same tendency was also detected for SSRs’ I values which superseded all the EST-SNP ones.

**Table 2:** Comparisons of genetic diversity parameters found by means of SSRs and EST-SNPs in the different WOGB core collections.

Core subsets	68		45		36		27		18	
Parameters/ markers	SSRs^∗^	SNPs	SSRs	SNPs	SSRs	SNPs	SSRs	SNPs	SSRs	SNPs
*N_avg_*	11.348	2.000	10.304	2.000	9.696	2.000	8.522	2.000	7.435	1.996
*N_e_*	4.127	1.544	4.188	1.550	4.367	1.547	4.078	1.548	3.967	1.553
*H_o_*	0.531	0.370	0.527	0.371	0.529	0.372	0.540	0.374	0.528	0.377
*H_e_*	0.686	0.329	0.692	0.332	0.696	0.330	0.698	0.330	0.693	0.332
*S_h_*	1.559	0.501	1.576	0.504	1.580	0.502	1.548	0.501	1.505	0.503


*^∗^The SSR values were taken from the original data set (Belaj et al., 2012). N_*avg*_, average number of alleles; N_*e*_, number of effective alleles; S_*h*_, Shannon’s Information Index; H_*o*_, Observed Heterozygosity; H_*e*_, Expected Heterozygosity.*

The EST-SNP analysis of 89 wild genotypes revealed that only 23 loci were monomorphic, and N_a_ and N_e_ were 1.98 and 1.44, respectively. In the examined wild germplasm, the mean I value was 0.42 and the average values of H_o_ and H_e_ heterozygosity were 0.27 and 0.28, respectively, with a PIC value of 0.23 (data not shown).

The One-way AMOVA showed that, although a great part of the genetic diversity was attributable to differences within wild and cultivated genotypes (61%), *ϕ_st_* value was significant (*p* < 0.001), suggesting the existence of genetic differentiation between them.

**FIGURE 2:**
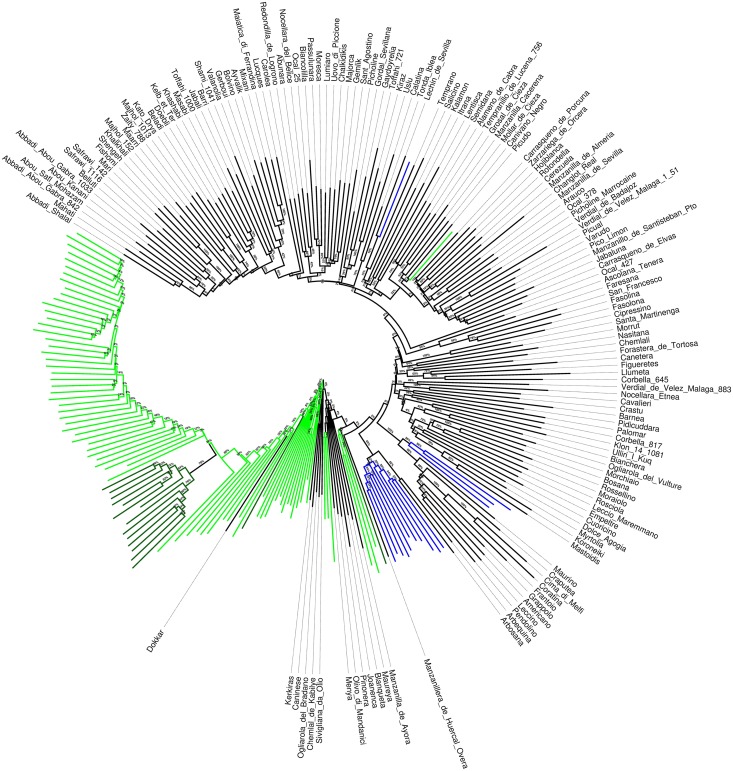
Neighbor joining dendrogram of wild olives, cultivated varieties and the genotypes from the cross ‘Picual’ x ‘Arbequina’ cultivars. Green branches correspond to wild olives, light green to the wild olives from Canary Islands, Blue branches refer to the crossbred progenies. Bootstrap values are reported as percentage at each branch node.

The relationships between cultivars, wild genotypes and crossbred progenies are presented in the NJ round tree (**Figure 2**). Cultivars and wild plants resulted into two clearly separated clusters, with a few exceptions of cultivars (such as ‘Dokkar,’ ‘Kerkiras’ ‘Chemlal de Kabilye,’ Sivigliana da Olio’ and ‘Ogliarola del Bradano’) included in the group of wilds while only one wild genotype (W168) was intermixed in the cultivated pool. The individuals of the inter-varietal cross progeny formed a clear subgroup within the cultivated varieties. Another subgroup was distinguished within the wilds and was represented by the samples from Canary Islands. Bootstrap values strongly supported the branching shaping of the dendrogram.

## Discussion

### EST-SNPs Variability

The results obtained in the present work represent a validation study of newly developed EST-SNP markers for olive germplasm identification. The experience acquired in the management of the olive germplasm collections and the progress in the knowledge on the richness of the olive genetic patrimony (Baldoni and Belaj, 2009; Bracci et al., 2011; Belaj et al., 2016), led our work to focus on the effectiveness of the new large set of EST-SNP markers to reliably discriminate among olive cultivars and to increase effectiveness and accuracy of germplasm collections.

The ability of reported EST-SNP markers as reliable tools to fully characterize the olive genotypes was evidenced by their wide variability, high number and easy scanning, comparable to that obtained by SNPs in other perennial species, such as grape (Myles et al., 2011), cacao (Zhou et al., 2016), longan (Wang et al., 2015), tea (Fang et al., 2014), citrus (Jiang et al., 2010), and litchi (Liu et al., 2015). The preliminary selection process of the most polymorphic SNP markers, as performed in previous studies (Biton et al., 2015), may explain the high values of genetic diversity obtained by assessing phylogenetic relationships in olive. In this sense, by maximizing the genetic diversity in a reduced number of genotypes (Belaj et al., 2012; El Bakkali et al., 2013), the core collections represent an ideal set of genotypes to validate the new EST-SNP markers. However, due to their biallelic nature, as opposed to the multi-allelism of microsatellites, the EST-SNPs displayed lower levels of diversity than SSRs on the same set of core collections examined. This potential drawback may be overcome by using a larger number of EST-SNP markers to reach the same level of discrimination (Biton et al., 2015; Zhou et al., 2016).

The pair wise comparison among the non-redundant cultivated genotypes (156) showed a high EST-SNP allelic differentiation. The 15 genotypes from the cross of ‘Picual’ x ‘Arbequina’ cultivars displayed similar allelic differentiation as the non-redundant cultivated genotypes, revealing thus high heterozygosity and low inbreeding within olive cultivars.

High ranges of allelic differentiation were also found for the wild genotypes, indicating a very high transferability of the new EST-SNP markers in the wild germplasm. Interestingly, diversity values in the wilds were lower than in cultivars probably due to crossing events between neighboring genotypes.

The genetic diversity within wild and cultivated genotypes clearly confirms the genetic differences between them, as already reported in previous studies by means of different markers (Baldoni et al., 2006; Belaj et al., 2007, 2010). Considering that these new set of SNPs are placed into EST, the differences pointed out between wild and cultivated genotypes will be thoroughly evaluated in future works for both evolutionary and functional aspects.

### Evaluation of the EST-SNPs Utility as a Means of Olive Germplasm Management

When setting up the new EST-SNP fingerprinting technique, one important concern was related to its reproducibility. On this respect, 100% concordance was found from multiple samples derived from independent DNA extractions of the same trees and/or different trees of the same cultivars. Besides, identical EST-SNP profiles were detected in some cases of previously identified or new introduced accessions, sharing the same names but coming from different plant sources and propagation events, i.e., doted by different register numbers. At the same time, in accordance to previous identification studies (Belaj et al., 2004b; Barranco et al., 2005; Atienza et al., 2013; Trujillo et al., 2014) by means of morphological descriptors and molecular markers (RAPDs, DArTs and SSRs), the same EST-SNP genotypes were found for the well-known redundant germplasm in the collection. These results validate the EST-SNP panel developed herein and fully agree with the high stability found by means of SNP markers in previous studies in olive (Biton et al., 2015), grape (Cabezas et al., 2011; Emanuelli et al., 2013) and coffee (Zhou et al., 2016). At the same time, our results demonstrate the usefulness of this new set of markers as a reliable tool for olive cultivar fingerprinting.

Most of olive varieties are traditional and can boast a multi- centennial origin, maintained over time in still alive trees (Díez et al., 2011; Barazani et al., 2014) or through vegetative propagation, and the presence of somatic mutations, with or without phenotypic effects, cannot be excluded (Martins-Lopes et al., 2009; Muzzalupo et al., 2010; Caruso et al., 2014; Lazović et al., 2016; Ninot et al., 2018). In fact, in numerous previous works, small SSR allelic differences have been attributed to the presence of clonal mutations (Cipriani et al., 2002; Martins-Lopes et al., 2009; Beghè et al., 2011; Díez et al., 2011; Caruso et al., 2014; Ninot et al., 2018). But the possible occurrence of uncertain interpretation of allele lengths has left the problem still open. Thus, consistent and reliable fingerprinting results are very important in long-living and clonally propagated species, where, although a low intra-cultivar polymorphism is expected, possible somatic mutation events may have occurred (El-Sharkawy et al., 2015; Zhang et al., 2017). In the present study, identical EST-SNP profiles were found for most cases of samples that had shown some slight differences when previously analyzed by other markers. This was the case of the cultivar pairs ‘Zarza’ – ‘Lechín de Sevilla,’ ‘Cordovil de Castelo Branco’ – ‘Verdial de Badajoz,’ ‘Picual de Hoja Clara-312’ – ‘Picual,’ ‘Picual de Hoja Oscura’ – ‘Picual’ (Trujillo et al., 2014), as well as ‘Frantoio A. Corsini’ – ‘Frantoio’ and ‘Moraiolo T. Corsini’ – ‘Moraiolo’ (Trujillo et al., 2014; Mousavi et al., 2017b). But, in other few cases (‘Picudo’ – ‘Picudo de Fruto Rojo,’ ‘Dulzal’ – ‘Manzanilla de Sevilla,’ and ‘Frantoio’ – ‘Razzola’ – ‘Maelia’), very small EST-SNP allelic differences were detected, reaching up to 0.005% (10 different alleles) of the total analyzed alleles. This value represents a very clear border between the highest intra-cultivar variability and the lowest inter-cultivar distance of 0.096% (200 different alleles) on total EST-SNP alleles. Similarly, very low intra-cultivar mutations rates have been revealed by the application of SNP markers in olive (Biton et al., 2015) and grape (Cabezas et al., 2011; Emanuelli et al., 2013). However, further analyses on the SNP genome location and sequence identity are needed to shed light on the origin of the SNP differences occurring in some genotypes. The use of new strategies of resequencing may be helpful to clarify at what rate mutations occur in perennial long living fruit crops as olive (Royo et al., 2015; Xie et al., 2016).

When using SSR markers, a less pronounced difference was found between possible intra-cultivar versus inter-cultivar variability (Trujillo et al., 2014), and/or a continue distribution (Haouane et al., 2011) of allelic differentiation between closely related and different genotypes. The clear cut-off between the highest and the lowest intra-cultivar variability obtained by EST-SNPs places them as the election markers for the solution of the numerous doubts on the identity of the analyzed genotypes. Previous studies in grape indicated that, even a lower but well-chosen set of SNP markers should allow to establish clear-cut boundaries to reliably distinguish between intra and inter-cultivar variation (Cabezas et al., 2011).

Another important issue within the olive genetic resources refer to well established synonyms, i.e., varieties with different names but identical molecular profile. In this sense, identical EST-SP profiles were observed both in previously described as well as in the new synonymy cases found among the cultivars in the present study. The existence of numerous cases of synonymy is the consequence of the millennial history of olive cultivation, which has favored a continuous migration and human displacement of olive cultivars among neighboring regions and countries, introducing cultivars into different regions and acquiring different names based on different local customs, tree bearing, fruit shape, etc. (Besnard et al., 2001; Khadari et al., 2008; Marra et al., 2013; Chalak et al., 2014; Ninot et al., 2018).

The presence of redundant genotypes, i.e., accessions that do not differ at all from the main profile of each cultivar, may occur frequently in olive germplasm banks (Atienza et al., 2013; Muzzalupo et al., 2014; Belaj et al., 2016; Mousavi et al., 2017b). The high efficiency of the new EST-SNP markers to ascertain the presence of duplicates is very important, as they represent a burden for curators and an increase in management costs, reducing the effective use of plant material. Besides, the EST-SNP identification of new accessions before their introduction into collection or before reaching their maturity, i.e., at early stages of tree growth, may be of great help for future agronomic evaluations, a time consuming and complicated task. In this sense, this set of EST-SNPs confirmed all cases of previously identified redundancies in the IFAPA WOGB collection (Barranco et al., 2000; Belaj et al., 2004b; Atienza et al., 2013; Trujillo et al., 2014). In addition to the presence of synonyms, the redundant genotypes found were mainly due to: (a) collection of redundant genotypes during the prospecting surveys, (b) introduction of potential intra-cultivar variants, and (c) possible errors (within and among accessions) in different stages of plant material introduction, conservation and management (Belaj et al., 2003a, 2004a; Barranco et al., 2005; Díez et al., 2011; Haouane et al., 2011; Dominguez-Garcia et al., 2012; Caruso et al., 2014; Chalak et al., 2014; Muzzalupo et al., 2014; Trujillo et al., 2014; Anestiadou et al., 2017; Ninot et al., 2018). Besides the confirmation of previously identified redundancies, the set of the EST-SNPs also allowed the detection of redundant germplasm among new and not previously assessed accessions. This may probably reflect the presence of duplicates within and between the regional donor collections, as well as possible management errors either at the receiving collection and/or at the collections of origin (Erre et al., 2010; Atienza et al., 2013; Marra et al., 2013; Muzzalupo et al., 2014). The presence of identical genotypes in different collections remains difficult to verify, until a common reference panel with reliable and discriminating markers will be applied. In this sense, the SNP panel developed in this work could further contribute to a thorough characterization of all olive cultivars and to establish a universal genotyping platform, allowing for an easy data comparison among different collections and laboratories.

The homonyms have usually been troublesome in olive cultivar identification as, historically, naming of cultivars have been based on common morphological traits (particularly of the fruit), toponyms or practical utility of the cultivars (Barranco et al., 2000; Belaj et al., 2016). The set of EST-SNP markers was very efficient at discriminating previously described homonymy cases demonstrating thus that generic names of cultivars (‘Abbadi,’ ‘Manzanilla,’ ‘Toffahi,’ ‘Carrasqueño’) include different genotypes (Barranco et al., 2000; Belaj et al., 2003a, 2004c; Trujillo et al., 2014). The use of different markers has shed light on the presence of many homonymy cases in olive germplasm collections (Noormohammadi et al., 2007; Fendri et al., 2010; Muzzalupo et al., 2014). Finally, 156 different olive cultivars from many olive growing countries were identified in the present study by means of EST-SNP markers, highlighting thus their utility in identification studies.

### Relationships Within and Among Cultivated and Wild Genotypes

In agreement with previous studies in olive with DArTs (Atienza et al., 2013), or EST-SSRs (Mariotti et al., 2016), the EST-SNP markers turned out to be easily transferable from the cultivated material to oleasters as well as to *O. europaea* subsp. *guanchica* genotypes. This is likely because they derived from conserved transcribed gene regions, shared among genomes of phylogenetically related species and subspecies (Myles et al., 2011). However, as reflected by various genetic parameters, the level of EST-SNPs genetic diversity detected in the wilds was lower than in the non-redundant cultivated germplasm, contrasting previous findings derived from SSR markers (Baldoni et al., 2006; Belaj et al., 2007, 2010; Erre et al., 2010). This result could be partly explained by the fact that the EST-SNPs used were selected for being polymorphic among cultivated genotypes.

In accordance to previous studies, (Baldoni et al., 2006; Hannachi et al., 2008; Belaj et al., 2010; Mousavi et al., 2017a), the EST-SNPs displayed a high genetic differentiation between wild and cultivated forms. Besides, the detection of a clear wild genetic background for some cultivars such as ‘Dokkar,’ ‘Chemlal de Kabilye’ and ‘Sivigliana da Olio,’ supports the introgression of local wild gene pools into domesticated olives (Belaj et al., 2010; Erre et al., 2010; Boucheffa et al., 2016; Mousavi et al., 2017a). At the same time, the similarity found between some wild and cultivated genotypes under study, may indicate a possible feral origin of some uncultivated genotypes (Belaj et al., 2010). Future studies by means of SNP markers based on wide and representative samples of wild and cultivated olives may shed further light on their genetic relationships and diversity.

## Conclusion

The high number of EST-SNPs developed in the present study, the relatively wide range of variability detected, their low intra-cultivar variation and genotyping error rates, and the fully automated procedure for the EST-SNP genotyping, indicate that they may become the markers of choice for olive germplasm identification, including both cultivars and wild plants. The availability of genome sequence data on cultivars (Cruz et al., 2016) and wild olives (Unver et al., 2017), as well as the continuous efforts to develop new SNP markers (Kaya et al., 2013; Biton et al., 2015; Ipek et al., 2016), will be of great help for such purpose. However, SSRs markers will still represent a good tool for a rapid cultivar identification, mainly when managing a small number of samples (Belaj et al., 2016).

In Conclusion, The present work represents a pilot study on the validation of EST-SNP markers for olive germplasm management with special attention to cultivar identification. The generated EST-SNPs showed a high variability in cultivated germplasm and also highly level of transferability to the wild relatives. The new EST-SNP markers revealed to be as efficient or even more than other molecular markers previously used for genotyping accessions at the IFAPA WOGB collection. The high number of EST-SNPs developed will allow the selection of an optimum core set of markers that will find a significant application for the identification studies in olive, to serve as a common and comparable set of markers for the entire olive research community. The olive DNA fingerprinting by means of a reliable panel of EST-SNP markers might represent a good opportunity to establish a universal and automated platform able to compare data across different laboratories, germplasm collections and genotyping platforms.

## Author Contributions

AB, RdlR, LB, and RM contributed to the conception and design of the work. AB and LB contributed to plant material collection. CB, OT, JG-P, AM-M, NC, and RM performed the molecular work including SNP mining and selection. AB, RdlR, RM, and IL contributed to genotype scoring, and data analysis,and interpretation. AB prepared the first draft of the manuscript. All the authors critically reviewed the manuscript prior to submission, read and approved the final version of the manuscript, and agreed to be accountable for accuracy, integrity, and appropriateness of the manuscript.

## Conflict of Interest Statement

The authors declare that the research was conducted in the absence of any commercial or financial relationships that could be construed as a potential conflict of interest.

## Footnotes

1 https://chirimoyo.ac.uma.es/oleagen/

2 http://www.oleagenome.org/

3 https://sourceforge.net/projects/seqclean/files/

4 http://tree.bio.ed.ac.uk/software/figtree/

## Supplementary Material

FIGURE S1 The NJ dendrogram including all cultivated plant material analyzed in the study.
