# Supplementary figures and images for: Usefulness of a New Large Set of High Throughput EST-SNP Markers as a Tool for Olive Germplasm Collection Management
Source: Front Plant Sci. 2018 Sep 21;9:1320. doi: 10.3389/fpls.2018.01320 (PMC6160578; doi:10.3389/fpls.2018.01320)

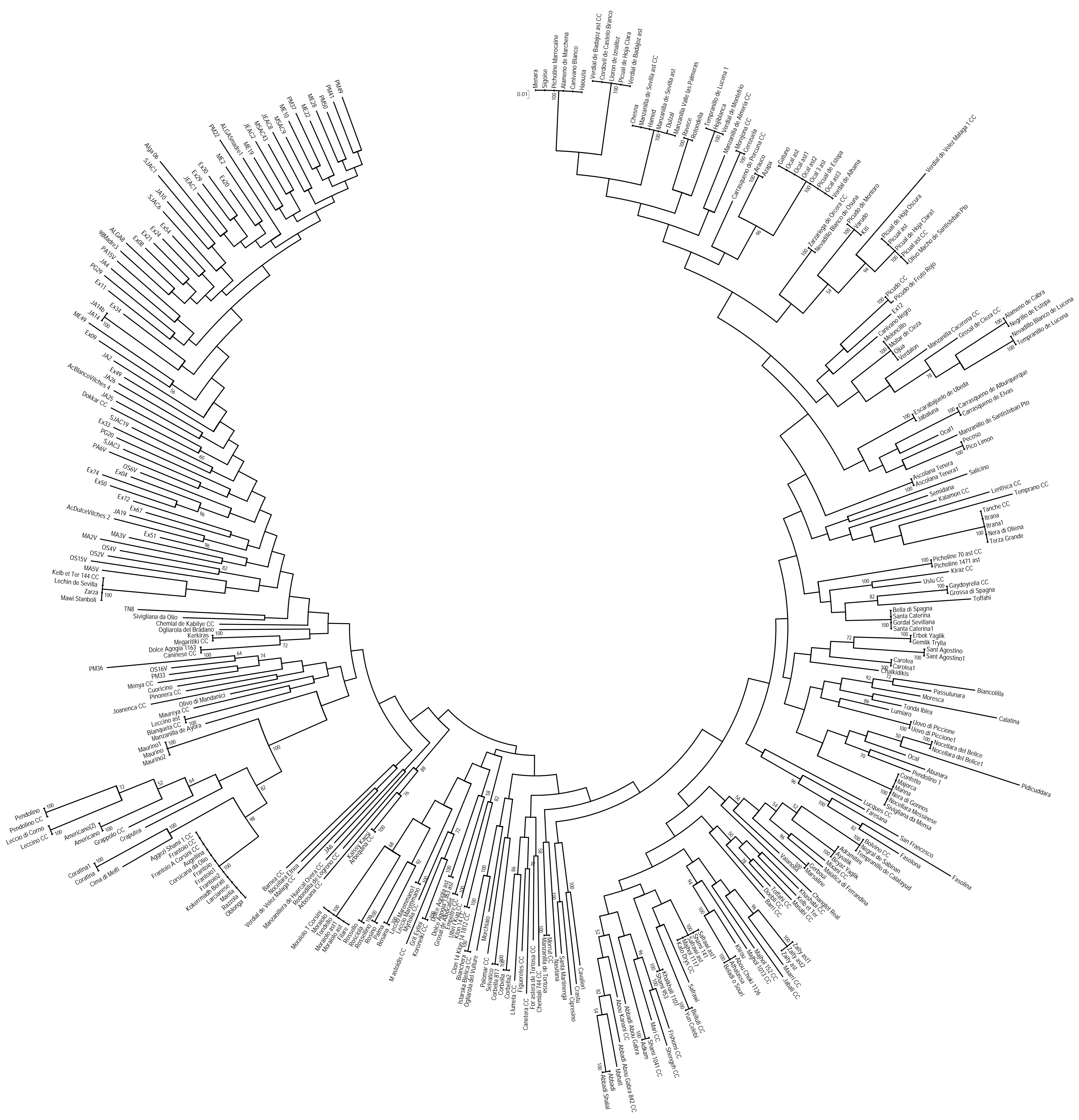

Supplement: FIGURE S1 — The NJ dendrogram including all cultivated plant material analyzed in the study. [file Image_1.PDF]
